# Formation of silicon nanowire packed films from metallurgical-grade silicon powder using a two-step metal-assisted chemical etching method

**DOI:** 10.1186/1556-276X-9-574

**Published:** 2014-10-14

**Authors:** Rachid Ouertani, Abderrahmen Hamdi, Chohdi Amri, Marouan Khalifa, Hatem Ezzaouia

**Affiliations:** 1 Laboratoire de Photovoltaïque, Centre de Recherches et des Technologies de l'Énergie, Technopôle de Borj-Cédria, BP 95, 2050 Hammam-Lif, Tunisie

**Keywords:** Silicon powder, Silicon nanowire, MACE, Grain size distribution, XRD, Raman spectroscopy

## Abstract

In this work, we use a two-step metal-assisted chemical etching method to produce films of silicon nanowires shaped in micrograins from metallurgical-grade polycrystalline silicon powder. The first step is an electroless plating process where the powder was dipped for few minutes in an aqueous solution of silver nitrite and hydrofluoric acid to permit Ag plating of the Si micrograins. During the second step, corresponding to silicon dissolution, we add a small quantity of hydrogen peroxide to the plating solution and we leave the samples to be etched for three various duration (30, 60, and 90 min). We try elucidating the mechanisms leading to the formation of silver clusters and silicon nanowires obtained at the end of the silver plating step and the silver-assisted silicon dissolution step, respectively. Scanning electron microscopy (SEM) micrographs revealed that the processed Si micrograins were covered with densely packed films of self-organized silicon nanowires. Some of these nanowires stand vertically, and some others tilt to the silicon micrograin facets. The thickness of the nanowire films increases from 0.2 to 10 μm with increasing etching time. Based on SEM characterizations, laser scattering estimations, X-ray diffraction (XRD) patterns, and Raman spectroscopy, we present a correlative study dealing with the effect of the silver-assisted etching process on the morphological and structural properties of the processed silicon nanowire films.

## Background

For almost two decades, the largest parts of silicon nanostructures that have been performed were porous silicon (pSi) using silicon wafers as a starting material. In recent years, the attention of several researchers and industrials has been gradually swerved from pSi to silicon nanowires (SiNWs). Indeed, SiNWs show noticeable advantages. Due to their good monocrystalline structure and electrical properties, SiNWs have been broadly explored for nanoscale electronic devices [1,2]. Microstructured silicon in wire shape is a good candidate to replace carbon lithium-ion batteries. SiNWs would be able to overcome problems caused by huge volume expansion during lithiation, enabling larger capacity and longer stability [3,4]. In addition to the excellent biodegradability and biocompatibility of silicon dioxide [5,6], SiNWs exhibit a relatively high surface-to-volume ratio. Hence, they could be readily oxidized and then functionalized with various biochemicals through different linkage chemistries. These properties have stimulated intensive researches attempting to evaluate the performance of SiNWs as biosensors and drug delivery systems [7,8]. It has been shown that porous SiNWs exhibit visible light emission and the luminescence is likely to be related to their porosity [9,10]. Improved NW porosity results in enhanced PL. Various synthetic methods have been reported to produce SiNWs. Bottom-up SiNWs are nearly totally grown from a high-purity silicon substrate for specific use in microelectronics and photonics. The bottom-up technique is expensive because it is time consuming and needs multistep fabrication and vacuum reactors (CVD, PLD, etc.) [11]. Even though metal-assisted chemical etching (MACE) is a top-down technique, which is rapid, simple, and of low cost, it relies on noble metal nanoparticles acting as catalysts in hydrofluoric acid (HF) solutions with an oxidant agent such as hydrogen peroxide (H_2_O_2_). Highly localized successive Si oxidation and etching take place underneath the metal nanoparticles. The etching procedure leads to the formation of a wirelike structure with a diameter close to that of the metal nanoparticle. SiNW morphology is affected by many factors such as the type of the semiconductor, orientation of the substrate, concentration of the oxidant, etching temperature, and etching time. Several papers and reviews reported the effect of those parameters and exposed in detail the methods as well as the chemical mechanisms that explain SiNW formation [12-15]. SiNWs sculpted in Si substrates may be collected by lift-off technique or by scratching the wires with a sharp blade followed by an additional grinding to obtain photosensitive nanoparticles. This method is simple but has a dramatically low output. To meet the huge demand of nanostructured silicon, a growing number of research teams are active to find new strategies and develop high-output batch processing capable of producing nanostructured silicon powder in quantities allowing upscaling to industrial demands [16-18]. So far, a low-cost and scalable method of silicon nanowire production remains a major challenge. This work is aiming to contribute to addressing this challenge. First, we chose silicon powder as the feedstock starting material instead of upgraded silicon wafers. This allows reducing nearly tenfold the price of the starting material. Practically, almost all SiNWs grown by the top-down approach were made from a monocrystalline silicon substrate. Very few works reported the processing of such nanostructure using silicon powder as a feedstock starting material [19,20]. Second, owing to the advantages offered by wirelike versus porous nanostructures, we preferred using a two-step MACE method instead of the widespread used stain etching in HF/nitric acid (HNO_3_) solution [21,22] by transferring MACE processing from Si wafers to Si powder. The starting samples are small amounts of metallurgical-grade polycrystalline silicon powder having 99.9% purity. We use silver as a metal catalyst, but other biocompatible metals and trace elements such as Fe and Mg could be experimented [20,23]. Many authors report synchronized anisotropic growth of SiNWs and branches of treelike silver dendrites during MACE [24-32]. However, some other authors report a simultaneous anisotropic etching of Si and isotropic growth of a fractal structure of silver clusters [24,25]. In this paper, we propose an explanation to the formation mechanism of rod-shaped overlapping silver microclusters. Besides, we highlight the role of the reagents in providing enough holes to facilitate the oxidation and the dissolution of the silicon atoms at the silver/silicon interface. Morphological and structural characterizations of the etched silicon powder have been conducted using scanning electron microscopy (SEM), X-ray diffraction (XRD), Raman spectra (RS), and a laser diffraction instrument (LDI). On the basis of these characterizations, we present a correlative study clearing up the SiNW film structure and morphology in connection to the etching duration.

## Methods

**Figure 1:**
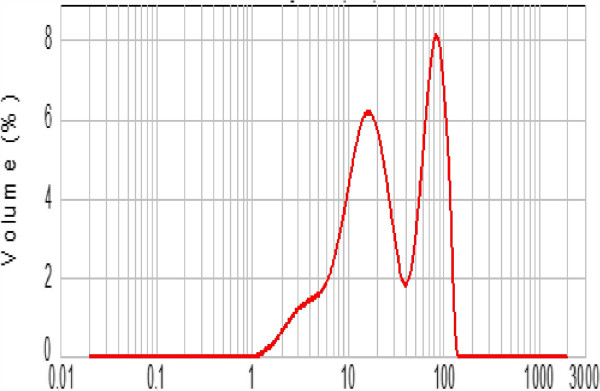
Grain size distribution of the untreated Si powder.

**Figure 2:**
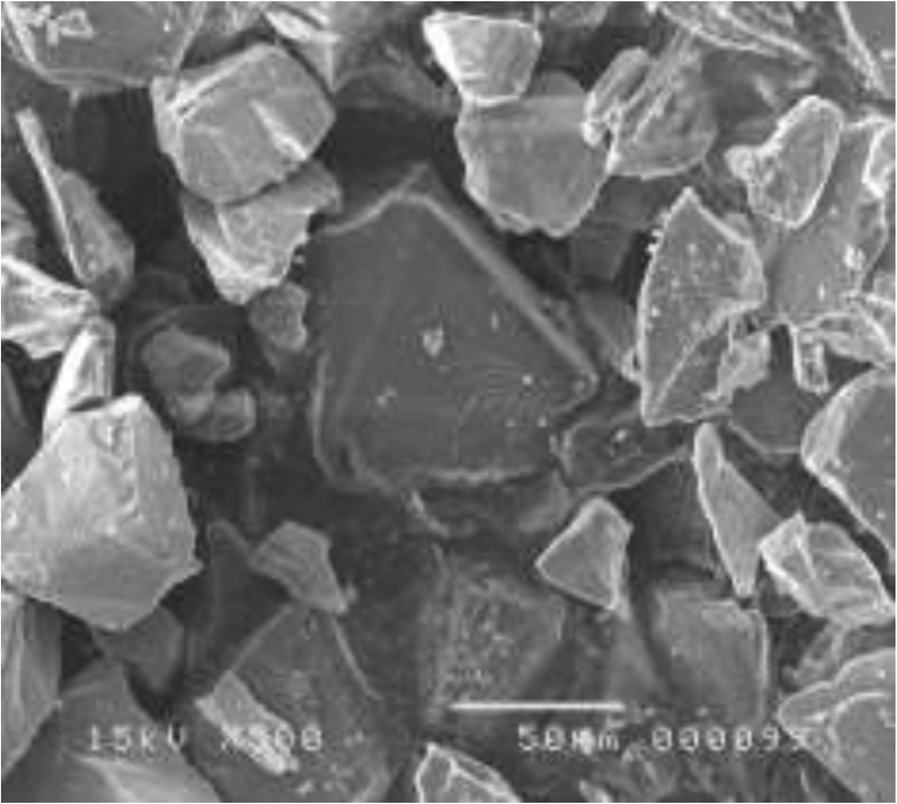
SEM micrograph of the starting powder.

The chemical reagents used in this work were hydrofluoric acid (HF; 40%), silver nitrate (AgNO_3_), hydrogen peroxide (H_2_O_2_; 30%), nitric acid (HNO_3_; 65%), and ethanol (C_2_H_5_OH; 65%). All of them were purchased from Sigma-Aldrich Corporation (St. Louis, MO, USA) and used without any purification, whereas metallurgical-grade polycrystalline silicon powder was obtained from Aremco Products Inc. (Valley Cottage, NY, USA). Prior to any treatment, the grain size distribution (GSD) of the received powder was measured with a Malvern Instruments Mastersizer 2000 (Malvern Instruments, Malvern, UK) using the laser scattering method. The powder is composed of polyhedral silicon micrograins (SiμGs) having a quite large grain size distribution. As depicted in Figure 1, the measurement has shown that the GSD of the starting Si powder ranges from 1 to 120 μm.Morphologies of silicon powder before and after chemical etching were characterized by SEM (JEOL JSM-5400, JEOL Ltd., Akishima-shi, Japan). Prior to SEM observation, a small amount of the treated Si powder was glued to a copper plate with a silver ink. SiμG dimension has been estimated from the SEM micrograph displayed in Figure 2. The values confirm in some way the Malvern Mastersizer measurements. It was revealed that the SiμGs have got random polyhedral shapes.

Inductively coupled plasma/atomic emission spectrometry (ICP-AES) reports that the raw Si powder is a quite ‘dirty’ dust with low purity (99.91%). Concentrations of the ten main impurities are presented in Table 1.

**Table 1:** ICP-AES analysis of impurities in raw Si powder

	**Element**									
	**Fe**	**Al**	**Ti**	**P**	**Ca**	**Na**	**Mn**	**Mg**	**K**	**Cr**
Impurity concentration (%)	5,100	2,200	421	16	98	38	793	55	20	230

Prior to the etching procedure, few grams of Si powder were degreased by successive treatments in acetone, ethanol, and de-ionized water and then dried at 50°C for 2 h.

**Figure 3:**
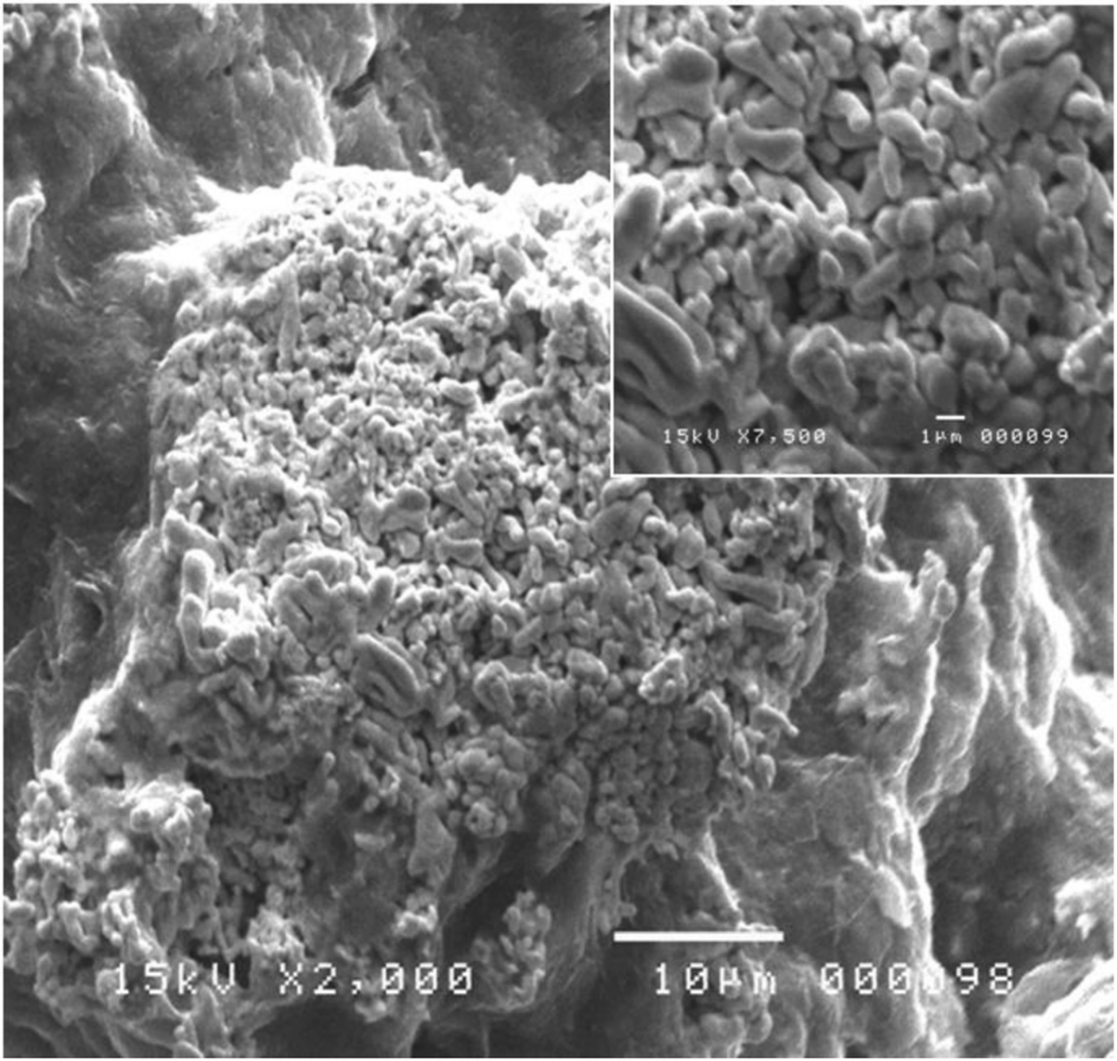
**SEM micrograph of Ag microclusters covering a silicon grain.** The insert shows a higher resolution SEM image highlighting the rodlike Ag microclusters.

In order to produce SiNWs out of silicon powder, we subject a small amount of the metallurgical-grade Si powder to an experimental procedure consisting of two consecutive steps. The first step is an Ag electroless plating process. The pre-cleaned powder samples were dispersed in tiny polyethylene pots containing 0.15 g AgNO_3_ and 4.6 M HF and then stirred for 5 min to permit the SiμGs to be covered with Ag. At the end of this step, a thick layer of Ag rodlike microclusters wholly wrapped the SiμGs as illustrated in Figure 3.

In the second step, MACE takes place by adding in the same beaker 0.12 M of H_2_O_2_. The silver-loaded clusters were left to be etched for a fixed duration. The reaction is exothermic; the solution temperature grew from room temperature to 70°C. As the etching reactions proceeded, gaseous by-products including hydrogen produced at the surface of the silver plating induced foaming of the powder. Consequently, most of the powder floated on the solution. This phenomenon has been witnessed earlier at least by four teams [3,18,20,22]. We adopt the same trick used by Loni et al. [20]. Therefore, ethanol was sprayed onto the foam to facilitate subsidence back into the acid solution. Even though this spraying operation was accompanied by a quenching of the reaction, it prevents partial etching of the Si powder. The reaction is practically stopped by adding a subsequent volume of de-ionized water. The etched powder was then soaked in a diluted nitric acid solution for 15 min to remove both clusters and residuals of silver. Finally, the samples were washed, filtered, and then dried at 50°C in air atmosphere before characterization.

## Results and discussion

**Figure 4:**
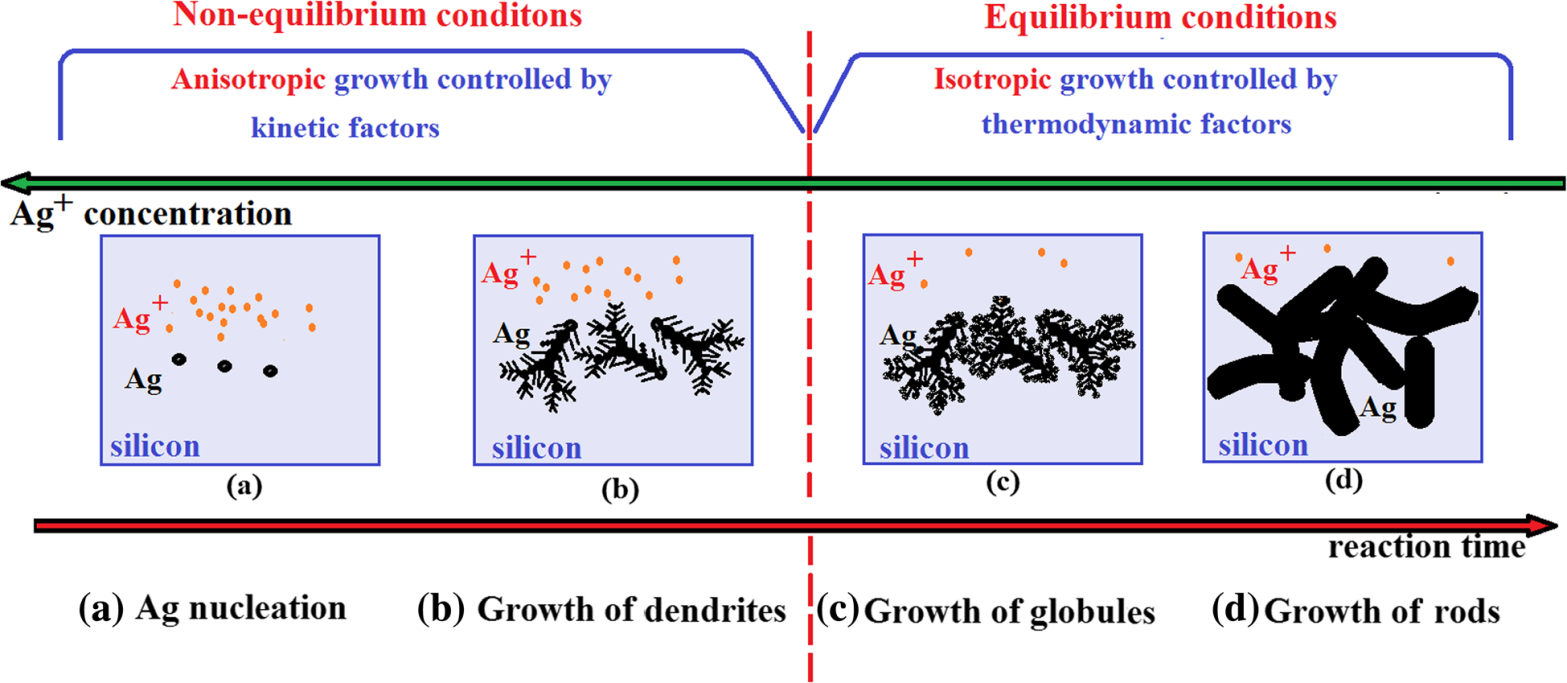
**Schematic illustration of the growth and evolution processes leading to the formation of rod-shaped silver microclusters. (a)** Ag nucleation. **(b)** Growth of dendrites. **(c)** Growth of globules. **(d)** Growth of rods.

**Figure 5:**
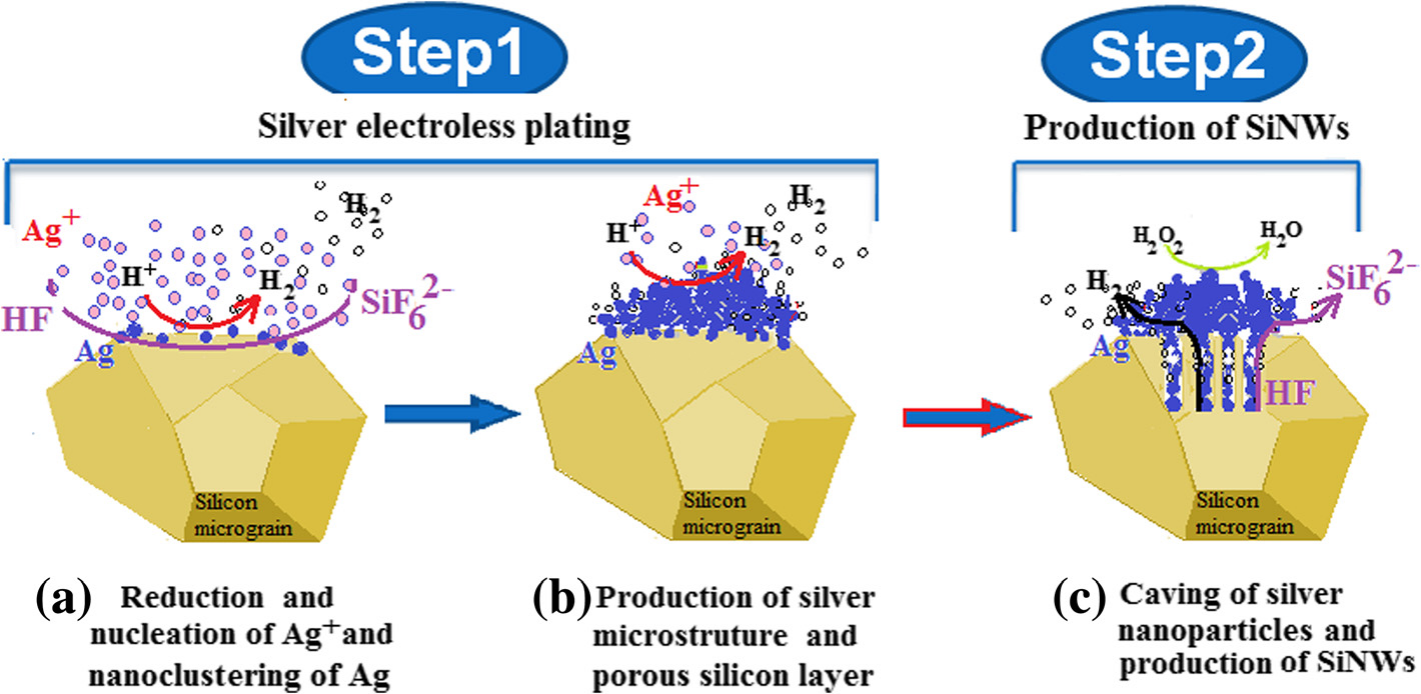
**Schematic illustration of the two-step MACE method leading to the formation of SiNWs from Si powder. (a)** Reduction and nucleation of Ag^+^ and nanoclustering of Ag. **(b)** Production of silver microstructure and porous silicon layer. **(c)** Caving of silver nanoparticles and production of SiNWs.

In this section, we try to elucidate the chemical mechanism occurring during each step of MACE. Silver microcluster growth and SINW formations will be highlighted. Illustrations in Figures 4 and 5 schematize the growth mechanisms leading to the formation of silver microclusters and SiNWs, respectively. Furthermore, we discuss the effect of the reaction duration on the morphology and structure of both silver clusters and silicon nanowire films. Finally, we propose a correlative explanation stressing the mutual effect between etching time, GSD evolution, XRD patterns, and Raman spectra.

### Silver deposition process

Silver plating of the SiμGs is the first step of the used MACE method (Figure 5). SiμGs have been completely plated by using the electroless silver deposition technique. This simple and inexpensive technique is based on a microelectrochemical redox reaction in which both the anodic and cathodic reactions occur simultaneously on the silicon surface. These reactions govern both silver thick film growth and porous SiNW formation. As the deposited silver films play the cathode role, silicon underneath them hosts the anode reactions [13-15]. At the end of the electroplating step, a thick layer of silver microstructure is produced on the surface substrates as displayed in the micrographs of Figure 3. A close observation to this micrograph reveals a fractal structure of rod-shaped microcluster units with rounded ends and no apparent necks. Previous works attempted to disclose both formation and evolution of silver dendrites into a fractal structure of globular patterns [14,24,25]. They report that silver structures grown in this process evolved from nanoparticles (or nanoclusters) to a dendritic pattern and then to a fractal pattern of dissymmetric microclusters. Similarly, we think that the formation of the obtained silver microclusters (Figure 3) can be explained as an evolution of the silver microstructure from treelike dendrites to rodlike networks during the electroless plating. The formation mechanisms of those microclusters can be schematically described by the illustrations of Figure 4.

Initially, the hydrofluoric acid solution contains a relatively high concentration of Ag ions. Thus, the reaction process is dominated by non-equilibrium conditions and the growth is controlled by kinetic factors. The initial stage is the nucleation phase which is initiated when the first silver ions come into contact with silicon atoms, take out electrons from the silicon valence band, and reduce to metallic Ag nuclei as schematized in Figure 4a. As the reaction proceeds, these nuclei grow up to larger nanoclusters negatively charged via Ostwald ripening [27]. Later on, nanoclusters grow via aggregation and coalescence effects [28] to form chain-like structures and then treelike morphologies distributed in a fractal structure, commonly called the dendrite structure, as depicted in Figure 4b. Many authors related that silver dendrite growth is anisotropic [29,30]. They found that the preferential growth directions are <100 > and <111>, leading to the formation of silver dendrites in those directions. Most of them believed that both global diffusion-limited aggregation (DLA) and oriented attachment of Ag nanoclusters are responsible for Ag treelike dendrite formation under non-equilibrium conditions [31]. As the galvanic displacement reaction proceeds, the Ag^+^ concentration should drop to such a level (less than 30 mM, according to Fang [32]) that the reaction process is dominated by equilibrium conditions. Therefore, the growth mechanism is no more kinetic but controlled by thermodynamic factors. A drastic transition of the silver aggregation patterns is observed. The initially feather-like or treelike morphology evolves gradually to a rod-shaped microstructure. Eventually, the feather tips transform gradually into round-shaped silver microclusters (Figure 4c). Both growth and coarsening of the globules are isotropic. Subsequently, the feather-like dendrites exhibited morphological evolution through tip coarsening to smooth silver rods grown in an isotropic manner as shown in Figure 4d. It has been established that a thick layer of silver clusters, but not dense, overlapping and covering completely the substrate promotes best the SiNW formation [14,33].

### Dissolution process

During the Ag plating step, simultaneously to the proliferation of the silver microstructures, there is a production of dihydrogen at the silver/HF electrolyte solution interface. The H_2_ production is accompanied by injection of holes to the silicon via the catalyst. On the other hand, each reduction of silver ions induces an excess of holes. These holes are injected into the valence band of silicon which oxidizes and then dissolves into silicon hexafluoride ions (SiF_6_^2-^) in the hydrofluoric acid aqueous solution. Throughout the dissolution process, Ag particles sink below towards the bulk silicon, creating irregular porous conic grooves roofed with Ag microstructures [15]. This synchronized mechanism of Ag^+^ reduction and silicon dissolution is illustrated in Figure 5 and labeled as step 1 corresponding to the silver plating process. The silicon dissolution process is likely to follow a microelectrochemical mechanism where the current flux is provided mainly by continuous silver ion reduction. Thus, the silver particles play the role of the cathode. Yet, silicon underneath the silver particle plays the role of the anode. The electrochemical redox reaction can be formulated as half-cell reactions (1), (2), and (3) [12,13,24,26,33,34]:

At the silver/electrolyte interface, the cathode reactions are described by Equations 1 and 2:

(1) $${Ag}^{+}\to Ag+h^{+}$$

and

(2) $$2\;H^{+}\to H_{2}+2\;h^{+}$$

At the silicon/silver interface, the anode reaction is given by Equation 3:

(3) $$Si+6\;F^{-}+4\;h^{+}\to {SiF}_{6}^{2-}$$

At a certain time, the hole injection rate becomes very slow because almost all Ag^+^ ions coming into contact with Ag particles or the silicon surface are reduced into Ag. Subsequently, silicon oxidation becomes very slow, causing a decrease in the silicon etching rate, hence the need to use a complementary hole injection species to allow the continuation of the silicon nanostructuring. Consequently, H_2_O_2_ is added to the etching solution because it is a strong oxidant capable of playing a major role of hole injection instead of Ag ions. The moment that coincides with the addition of the hydrogen peroxide is the start time of the second step related to the MACE reactions as it is illustrated in Figure 5 under the label step 2. In fact, Si atoms underneath Ag particles receive holes not only from the reduction of hydronium ions (H_3_O^+^) but mainly from the reduction of hydrogen peroxide (H_2_O_2_). Both reactions occur at the silver cluster/acid solution interface. In brief, H_2_O_2_ contributes greatly in increasing the hole flow rate towards silicon and facilitates its dissolution. Furthermore, H_2_O_2_ may even oxidize the Ag particles previously nucleated on the silicon surface or stacked to the silver microclusters. This oxidation permits the feeding of the solution with Ag^+^ ions, enhances hole injection, and keeps the silicon dissolution going. Both silver microclusters and silicon surfaces maintain their microelectrochemical roles as during the silver plating step: cathode and anode, respectively. Cathode and anode reactions are sketched and exposed by numerous previous works [12,13,24,33,34]. Among the numerous models proposed for the dissolution process of silicon, we think that the model established by Chartier et al. is the most convincing. Indeed, in contrast to other models, Chartier and co-workers evidenced that H_2_ is produced as an anodic reaction simultaneously to the direct dissolution of silicon in its divalent state (SiF_6_^2-^) [13]. This process can be described by the following equations:

At the silver/electrolyte interface, the cathode reaction is described by Equation 4:

(4) $$H_{2}O_{2}+2\;H^{+}\to 2\;H_{2}O+2\;h^{+}$$

At the silicon/silver interface, the anode reaction is given by Equation 5:

(5) $$Si+6\;HF+3\;h^{+}\to H_{2}{SiF}_{6}+3\;H^{+}+\frac{1}{2}\;H_{2}$$

The overall reaction can be represented by the following Equation 6:

(6) $$2\;Si+12\;HF+3\;H_{2}O_{2}\to 2\;H_{2}{SiF}_{6}+3\;H_{2}O+H_{2}$$

Therefore, during silicon dissolution, H_2_ is generated. H_2_ bubbles provoke the foaming of the powder and prevent homogenous etching of the whole powder. As it has been mentioned above, we overcome this hindrance by spraying the foam with ethanol.

According to a recent work of Hildreth et al. [35], van der Waals forces are the possible driving forces behind the catalyst (Ag) caving inside the silicon during MACE. It is worth noting that the etching direction depends on the substrate orientation, the particle shape of the catalyst, and the relative concentration of the reagents [12,33].

### Correlation between morphology, structure, and grain size distribution

**Figure 6:**
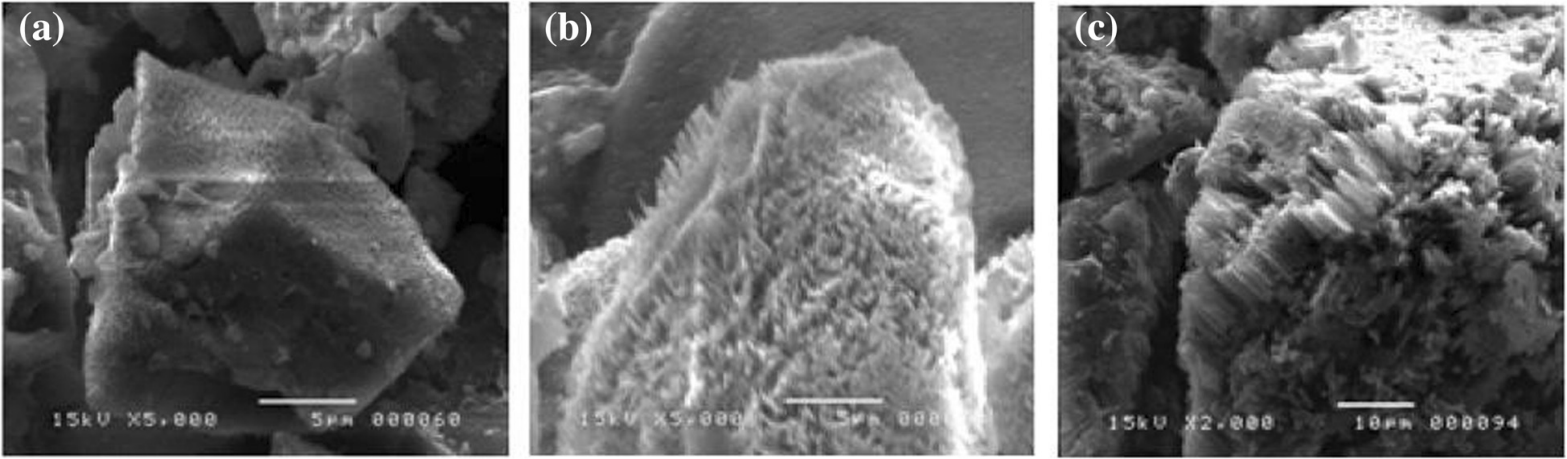
SEM images of MAC etched Si powder for (a) 30 min, (b) 60 min, and (c) 90 min.

**Figure 7:**
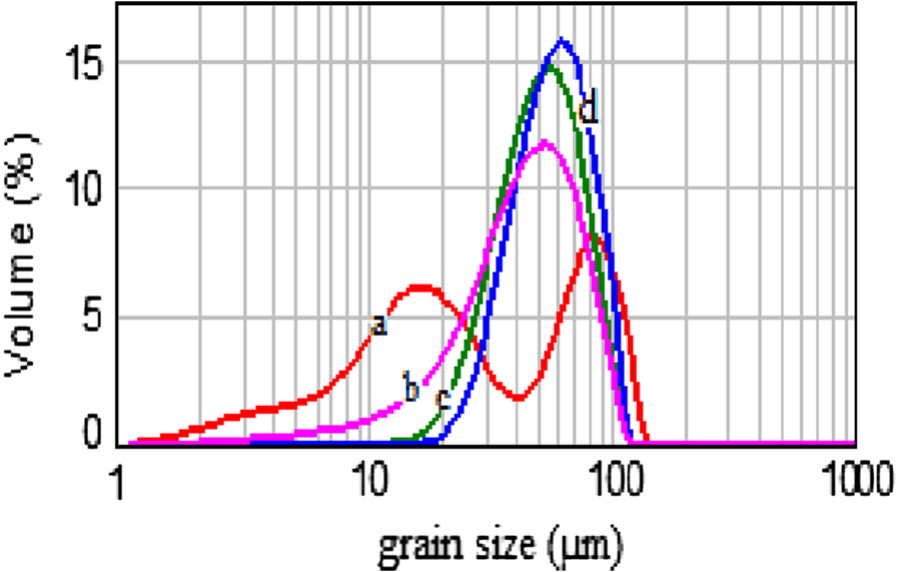
GSD of MAC etched Si powder for (a) 0 min, (b) 30 min, (c) 60 min, and (d) 90 min.

**Figure 8:**
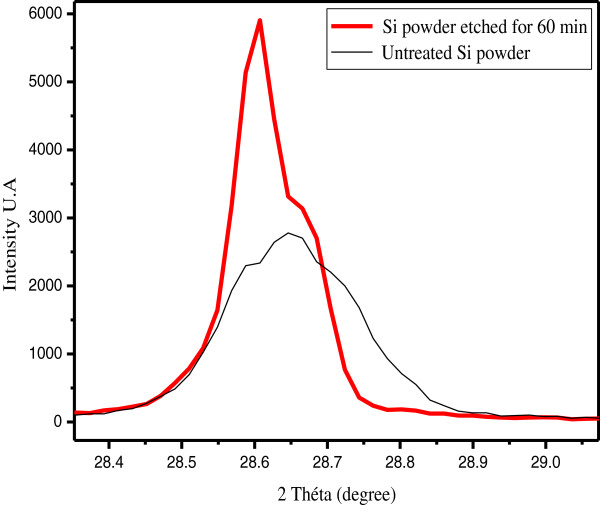
X-ray diffraction diagram of the untreated raw Si powder and Si powder after 60-min MACE.

Three samples were left for etching times of 30, 60, and 90 min. SEM micrographs of these samples are displayed in Figure 6. They reveal that all facets of the SiμGs are covered with a densely packed film of self-organized nanowires. Some SiNWs were perpendicular to the facets but others slanted to the SiμG surfaces.According to these micrographs, after 30 min, nanosized Si pinecones began to appear. Some of them reach 0.2-μm height. As the etching time increases to 60 min, more nanowires were formed having 2.5-μm height. The average diameter of the wires is approximately 100 nm. After 90 min, Si pinecones disappeared whereas the SiNWs appeared taller, having 10-μm height. However many SiNWs were broken; some others congregated together (Figure 6c). The increasing etching rate observed in the last sample corresponding to 90-min etching time might be attributed first to the high density of defects in the starting SiμGs and then to the complete dissolution of the small-sized grains as a consequence of the decrease in the total surface area of the powder. Figure 7 depicts the etching time effect on the grain size distribution of the powder.Indeed, each etched SiμG is composed of a solid core covered by a densely packed film of SiNWs. A short etching duration ensures only the formation of a shallow thin film of SiNWs with thicknesses not exceeding 10 μm. Nevertheless, too long etching leads to a complete dissolution of the small SiμG. Since the initial Si powder consists of SiμG with a wide range of sizes (from 1 to 120 μm), only a part of them could be more or less partially nanostructured. It is obviously expected that the etching process would dissolve completely all silicon grains having a size dimension smaller than twice of the SiNW height. On the other hand, grains having a random shape with disparate sizes in two or three dimensions, simply, become smaller. SiμGs whose size exceeds 20 μm keep their size practically stable. This effect is confirmed by the GSD patterns displayed in Figure 7. Grain size frequencies shift towards medium-sized grains. Accordingly, the metal-assisted chemical etching narrows the GSD of the starting powder.On the basis of the XRD patterns in Figure 8, one may state that the lattice structure of the nanowires is nearly identical to that of bulk silicon.

As it is suggested by the XRD patterns, the Si powder is polycrystalline with a facet having various crystallographic orientations. We note that (111), (220), and (311) are the crystallographic orientations corresponding to the main surface facets present in the powder.

**Figure 9:**
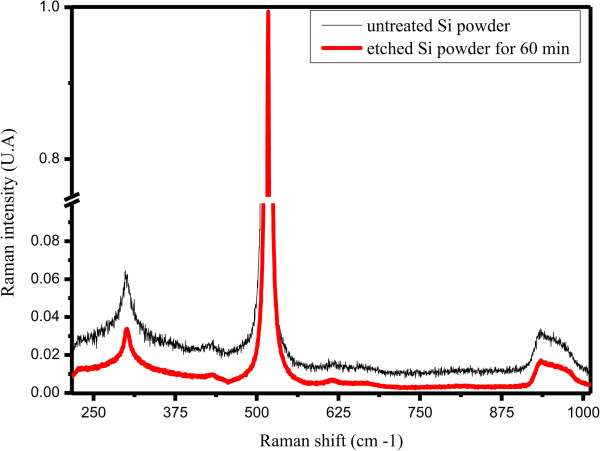
Peaks corresponding to the (111) plane in Si powder before and after MACE.

The various nanowire directions observed in SiμGs are in good agreement with the anisotropic behavior of MACE. Indeed, due to the different back-bond strength, the Si atom on the (100) surface plane is the most easily removed, and the etching occurs preferentially along the <100 > direction. The weaker is the back-bond strength, the easier it is to remove a silicon atom. The number of back-bonds of an atom on a plane is determined by the crystallographic orientation of the plane. For instance, each atom on the (220) or (111) surface plane has three back-bonds while the (100) surface plane has only two back-bonds [12].However, the full width at half maximum of the XRD peaks of the Si nanowires is about 2.5 times narrower than the corresponding one of the reference Si powder. Figure 9 shows a comparison between two peaks corresponding to the (111) plane in raw Si and SiNW samples.

**Figure 10:**
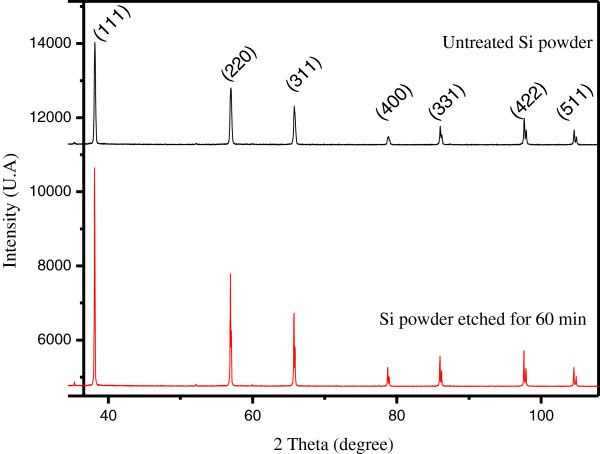
Raman spectra of Si powder before and after MACE.

Using the Scherrer equation, we found that the average dimension of the ordered crystalline domains moves from 60 to 150 nm. We may attribute this noticeable enhancement in crystalline structure to three main reasons. First, the raw powder was initially covered by a native amorphous silicon dioxide which enlarges the corresponding XRD patterns. HF acid, being part of the etching solution, dissolves the silicon dioxide during the etching process and reveals the crystalline bulk structure of the grains. Second, as it has been explained above, MACE contributed to narrowing the grain size distribution by dissolving small SiμGs. Afterward, the XRD patterns were slenderer, indicating an apparent improvement of the overall crystalline structure of the powder. The third reason is the purification effect of the MACE processing induced by the dissolution of the metal impurities and crystal defects initially present in the starting raw silicon powder.Figure 10 shows two RS having similar patterns.

The upper one corresponds to the raw Si powder and the other one to the SiNW sample. RS of the raw Si powder is similar to a typical spectrum of monocrystalline silicon. It appears that each RS has three main peaks. The most intense central peak corresponds to the first-order phonon mode, an optical active mode triply degenerated. On either side of the central peak, we observe two less intense peaks corresponding to the spectrum of second-order Raman spectra involving two phonons. The midpeak has a frequency of about 516.8 cm^-1^ instead of the typical 520 cm^-1^ of silicon. This peak is much thinner, almost 57 times more intense than raw Si. This notable Raman peak shift towards higher energy might be attributed to the same three reasons mentioned above to explain the XRD pattern variations. Indeed, both metal impurities and amorphous silicon oxide initially present inside and at the surface of raw silicon powder disturb the Si crystalline structure and induce tensile strains, respectively. Therefore, the etching process smoothes the tensile strains and enhances the crystalline structure. On the basis of the recent work of Khorasaninejad et al., we can partially attribute this enhancement in the Raman scattering intensity to the increased confinement of light within the wires [36].

Correlation between GSD, XRD, and RS patterns performed on silicon powder before and after MACE treatment shows the disappearance of SiμGs whose size is smaller than few tens of microns while the SEM micrographs demonstrate an increase in nanowire length. This proves that the SiNWs are indeed attached to the large SiμG. The wires should have the same crystalline structure. Otherwise, tensile strains at the SiNW/Si bulk interface would induce constraints that promote defects leading to a locally high silicon dissolution rate. As a result of this hypothesis, the SiNWs would either break or disappear completely in the solution during the etching process. This hypothesis lapses because it is contrary to SEM observations and Raman spectra. Besides, XRD analysis shows that the MACE treatment of the powder causes a reduction of tensile strains within the starting powder. Therefore, we can conclude that the silicon nanowires are monocrystalline and have a similar crystal structure to the facet in which they are grooved. As it has been mentioned above, MACE is anisotropic, so the wires stand vertically or tilt to the surface facets of the silicon grains.

## Conclusion

This work deals with the development of a rapid, cost-effective, and scalable process to fabricate silicon nanowire-covered micrograins (μGs) from a cheap metallurgical-grade polycrystalline silicon powder. We transfer a two-step MACE method from Si wafers to Si powder. We highlighted the growth mechanisms leading to the formation of silver microclusters and SiNWs during the electroless Ag plating and the silicon grooving steps. SEM micrographs showed that the two-step MACE method enabled the grooving of densely packed films of SiNWs having lengths ranging from 0.2 to 10 μm. On the basis of XRD patterns and Raman spectroscopy, we showed that the nanowires were perfectly crystalline, oriented perpendicularly or tilted to the facets of the SiμGs. We evidenced that MACE enhances the apparent crystalline structure of the Si powder. We attribute this enhancement to the removal of both native amorphous silicon dioxide and atom impurities initially present in the starting raw metallurgical-grade silicon powder.

## Abbreviations

GSD: grain size distribution; MACE: metal-assisted chemical etching; RS: Raman spectra; SEM: scanning electron microscopy; SiNWs: silicon nanowires; XRD: X-ray diffraction; μGs: micrograins.

## Competing interests

The authors declare that they have no competing interests.

## Authors' contributions

OR analyzed the experimental results, proposed a correlative interpretation, and drafted the manuscript. HA handled the Si powder, performed the chemical etching, and helped in the structure characterization. AC performed the SEM observation. KM performed the characterization of the raw silicon powder with ICP-AES. EH directed the overall study and discussed the interpretation of the results. All authors read and approved the final manuscript.

## Authors' information

OR is an assistant professor. HA and AC are Ph.D. students. MK is a researcher. Finally, EH is a professor. All authors are in the Laboratory for Photovoltaic at the ‘Centre de Recherches et des Technologies de l'Énergie’ (CRTEn).
